# Hemoculture and Polymerase Chain Reaction Using Primers TCZ1/TCZ2 for the Diagnosis of Canine and Feline Trypanosomiasis

**DOI:** 10.5402/2012/419378

**Published:** 2012-05-31

**Authors:** Luciano José Eloy, Simone Baldini Lucheis

**Affiliations:** ^1^Departamento de Doenças Tropicais e Diagnóstico por Imagem, Faculdade de Medicina de Botucatu (FMB), Universidade Estadual Paulista (UNESP), 18618970 Botucatu, SP, Brazil; ^2^Agência Paulista de Tecnologia dos Agronegócios (APTA) Pólo Centro Oeste, 17030-000 Bauru, SP, Brazil

## Abstract

*Introduction*. American trypanosomiasis, also known as Chagas disease, is a zoonosis caused by *Trypanosoma cruzi* (*T. cruzi*). Dogs and cats participate actively in this parasite's transmission cycle. This study aimed at evaluating the occurrence of *T. cruzi* in dogs and cats from Botucatu, SP, Brazil, as well as at evaluating the technique of hemoculture in LIT (liver infusion tryptose) medium by polymerase chain reaction (PCR). *Methods*. Blood samples were collected from 50 dogs and 50 cats in Botucatu-SP, Brazil. For hemoculture, the samples were inoculated in LIT medium, and readings were performed for four months. Upon completion of such period, all the hemocultures were processed for parasitic DNA extraction. The PCR reactions were performed by using primers TCZ1/TCZ2. *Results*. Ten dogs and ten cats (20%) were positive to PCR, and four dogs and three cats (7%) were positive to hemoculture. Only in a one cat sample (1%) there was confirmation of positive hemoculture by PCR for *T. cruzi*. *Conclusions*. Results showed that PCR was a suitable tool for the confirmation of the parasite detection in hemoculture samples, and that dogs and cats from Botucatu, SP, Brazil, are maintaining the role of household reservoirs of *T. cruzi*, which reinforces the need for constant epidemiologic surveillance for this zoonosis.

## 1. Introduction

American trypanosomiasis, also known as Chagas disease (CD), is an important zoonosis, and its etiologic agent is the flagellate protozoan *Trypanosoma cruzi* (*T. cruzi*) [1]. In Brazil, according to serological survey carried out between 2001 and 2008 in all states, it is estimated that approximately 133,000 people are infected with *T. cruzi* [2]. Originally, American trypanosomiasis was an enzootic disease as it affected only wild mammals. Today, *T. cruzi* has been detected also in domestic mammals such as dogs and cats, thus making trypanosomiasis a typical zoonosis [3]. Dogs and cats infected with *T. cruzi* act as infection sources for humans and other animals, thus being important reservoirs of trypanosomatids among household animals [4].

 Hemoculture, which consists in the enrichment of a blood sample, thus enabling the multiplication of existing parasites, represents an indirect parasitological test used in the chronic phase of CD. Positive hemoculture, when utilized for diagnose, shows the presence of the parasite in the blood stream. This is a limitation of the hemoculture technique when it is compared at polymerase chain reaction (PCR) technique, which can detect parasite fragments and does not require the presence of whole organisms in the blood stream [5].

The serological and parasitological assays, such as hemoculture, cannot differentiate *T. cruzi* from other trypanosomes [6], such as* Trypanosoma rangeli (T. rangeli), *that can infect various mammal species, including humans [7]. TCZ1/TCZ2 primers, utilized at PCR technique for detection of specific DNA of *T. cruzi*, are capable of amplifying all *T. cruzi *lineages [8] and not amplifying the DNA of another trypanosome [9].

TCZ1/TCZ2 primers amplify a repetitive sequence of a microsatellite region of the nuclear DNA (nDNA) of *T. cruzi* [9]. This region is the miniexon gene, which presents a nuclear location that is relatively conserved among species of the same genus, showing distinct nucleotide sizes and sequences even among very close species [10].

Hence, the present work aimed at studying the occurrence of *Trypanosoma cruzi* in cats and dogs from the city of Botucatu, São Paulo (SP), Brazil, as well as at evaluating the hemoculture technique in LIT (liver infusion tryptose) medium by the polymerase chain reaction (PCR), using TCZ1/TCZ2 primers for *T. cruzi* detection.

## 2. Methods

### 2.1. Studied Animals

One hundred animals were researched, being 50 dogs (23 males and 27 females) and 50 cats (18 males and 32 females). Thus, this study researched 41 males and 59 females.

### 2.2. Blood Samples and Collection

The blood samples were randomly collected at the Municipal Kennel and Animal Protection Association (APA) of Botucatu, SP, Brazil. Five mL to eight mL of blood were collected from each animal by means of a jugular venous puncture using tubes containing EDTA. The samples were cooled and immediately sent to the laboratory, where were processed for hemoculture.

### 2.3. Sample Processing and Hemoculture Technique Performance

 The processing of the samples collected from the animals at the Municipal Kennel of APA in Botucatu-SP, Brazil, was performed at the Center for Zoonosis Research-NUPEZO of the Department of Veterinary Hygiene and Public Health of the Botucatu School of Veterinary Medicine and Animal Husbandry (UNESP) in São Paulo state, Brazil, and in the Laboratory of Tropical Diseases of the Department of Tropical Diseases and Imaging Diagnosis of the Botucatu School of Medicine (UNESP) in São Paulo state, Brazil.

### 2.4. Hemoculture in LIT Medium

The medium used for blood culture was Liver Infusion Tryptose (LIT). In order to prepare 250 mL of this medium, 2 g of Na_2_HPO_4_ (Nuclear), 1 g of NaCl (Synth), 0.1 g of KCl (Dinâmica), and 44.5 mL of Milli-Q water were placed in a sterile Kitasato flask. A stainless-steel filter holder (Millipore), with a membrane whose pore size was 22 *μ*m (Millipore), was adapted to the Kitasato flask, sealed with aluminum foil and manila paper and autoclaved at 121°C for 20 minutes. Simultaneously, 0.75 g of liver infusion (Difco) were diluted in 100 mL of heated Milli-Q water, 0.5 g of Dextrose (Oxoid), and 1.25 g of Tryptose (Vetec^®^), setting the solution pH at 7.4. Such dilution was filtered in the Kitasato flask, which was coupled to a vacuum pump. Later, 5.5 mL of Bovine hemoglobin were filtered at 2.2% (BBLTM). In the laminar flow hood, 30 mL of fetal bovine serum at 11% (Nutricell) and 1 mL (20 mg) of gentamicin (Gentocin) were added in sterile conditions. Finally, sterility control was performed in a sterilizer at 37°C for 48 h using BHI medium. The final content was distributed in sterile tubes with threaded caps and maintained in a stove at 28°C–30°C until the moment of use.

For every blood sample collected, one sterile threaded tube was separated, each one containing five mL of the sterile LIT medium. The blood samples were handled in a laminar flow hood. Next, the cultures were incubated and maintained in an incubator at 28°C–30°C up to four months after inoculation, and then submitted to the Polymerase Chain Reaction technique (PCR) for *T. cruzi.*

### 2.5. Hemoculture Reading

The first reading was performed ten days after the inoculation in LIT medium of the first sample by removing five microliters from each tube of inoculated culture and placing it between a slide and coverslip. Five slides per tube were read fortnightly for four months. Readings were performed on a light microscope at 1.000x magnification using immersion oil. The positive cultures were immediately processed for extraction of the parasitic DNA. The negative cultures were also performed upon completion of the four-month period of reading.

### 2.6. Preparation of Blood Samples in LIT Medium for Parasitic DNA Extraction

Both the positive and negative cultures were separately washed in sterile phosphate-buffered saline solution, 0.01 M (pH 7.2) and centrifuged at 1000 rpm for 10 minutes, and the sediment was stored in sterile-free tubes DNAse and RNAse at −20°C until the moment of parasitic DNA extraction [11].

### 2.7. DNA Extraction

DNA was extracted from 300 *μ*L of the stored sediment by using Illustra Blood Genomic Prep Mini Spin kit (GE Healthcare). It was later stored in sterile free tubes DNAse and RNAse and maintained at −20°C until the moment of PCR test.

### 2.8. Polymerase Chain Reaction for *Trypanosoma cruzi*

 TCZ1 (5′-CGAGCTCTTGCCCACACGGGTGCT-3′) and TCZ2 (5′-CCTCCAAGCAGCGGATAGTTCAGG-3′) primers were used, since they are species-specific for *T. cruzi* and amplify an nDNA microsatellite region of 188 base pairs [9, 12].

All reactions were performed in duplicate, containing 2.5 *μ*L of PCR buffer (50 mmol KCl, 10 mmol of Tris-HCl, and 1.5 mM MgCl_2_), 0.2 mM of each deoxynucleotide triphosphate, 1.0 U of *Taq*-polymerase, 10 pmol of each primer, 2 *μ*L of DNA, and 17.8 *μ*L of ultrapure water for a final volume of 25 *μ*L. The conditions of amplification in a thermocycler (Mastercycler Personal, Eppendorf) occurred according to Virreira et al. [12] as follows: one cycle for initial denaturation at 94°C for 5 minutes; 35 amplification cycles (denaturation) at 94°C for 20 seconds, annealing at 57°C for 10 seconds and extension at 72°C for 30 seconds, and one cycle of 72°C for seven minutes for terminal extension, respectively.

The 10 *μ*L aliquots of the samples amplified by using primers TCZ1/TCZ2 were homogenized with 2 *μ*L of bromophenol blue solution, submitted to horizontal agarose gel electrophoresis at 10% in 10x tris-borate-EDTA (TBE) buffer and stained by Gelred (Biotium, Inc.) for identification of the amplified products. The run was performed by using the same TBE buffer at 80 volts for 100 minutes, and the bands were viewed on an ultraviolet transilluminator (Benchtop M-20-UVP). The brand of the electrophoresis chamber used was Uniscience.

*Trypanosoma cruzi* Y-strain was used as a positive control, MIX-PCR as negative control, and 3 *μ*L of DNA Ladder (NORGEN) 100 bp were utilized as a molecular weight standard.

## 3. Results

### 3.1. Hemoculture

Of the 50 dogs, four samples (8%) showed viable parasites in the culture and, of the 50 samples from cats, parasitic forms were observed in three (6%).

Among the dogs, one sample was diagnosed in the first reading (10 days after inoculation), two were found 25 days after inoculation and one after 40 days. In the group of cats, the positive samples were diagnosed in the first reading.

### 3.2. Polymerase Chain Reaction

**Figure 1:**
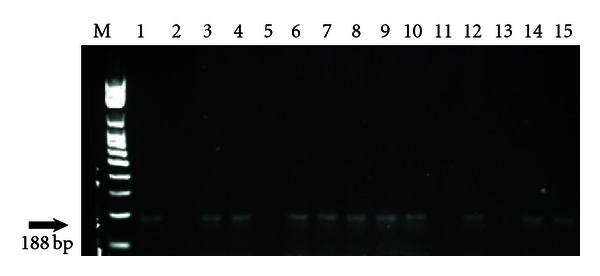
1.0% agarose gel stained by GeLred. Amplification of fragments of 188 bp of *Trypanosoma cruzi* by PCR, using primers TCZ1/TCZ2 in 13 hemoculture samples for dogs and cats from Botucatu, SP. M: Standard molecular weight for DNA (100 bp); 1: positive control (*T. cruzi-*Y strain); 2: negative control (MIX PCR); 3: dog 40; 4: dog 41; 5: dog 10; 6: cat 32; 7: cat 35; 8: cat 44; 9: cat 36; 10: dog 46; 11: dog 01; 12: cat 39; 13: cat 45; 14: cat 49; 15: dog 50, botucatu, SP, Brazil.

Of the 50 dogs evaluated, ten (20%) were positive, and regarding the 50 cats, ten (20%) showed amplified products of 188 base pairs (bp) in length (Figure 1).

### 3.3. Hemoculture and Polymerase Chain Reaction

Of the 100 animals evaluated, just one cat (1%) was positive for hemoculture and PCR (Table 1).

### 3.4. Gender of the Animals

 Of the 59 females of this study, only three (5.08%) were positive for hemoculture. Among the males (41), four (9.75%) animals were positive for at least one of two techniques, being only a cat positive for both techniques (Table 1).

### 3.5. Statistical Analysis

The statistical analysis showed that the hemoculture technique presented low sensitivity—14% (0.14) and specificity in 89% (0.89) to identify trypanosomatids, beyond relative sensitivity and specificity (i.e., sensitivity and specificity in relation to PCR), respectively of 14% (0.14) and 79% (0.79). This postulation is confirmed by the positive likelihood ratio (1.64), showing that hemoculture identifies many false negatives, 64%, and by the negative likelihood ratio (1.04), showing that hemoculture has 96% of chances to identify negative samples, that is, it identifies almost only negative samples.

 Additionally, the positive predictive value (0.05) showed that the hemoculture technique presented small capacity to predict positives, only 5%, and the negative predictive value (0.92) demonstrated great capacity to predict negatives (92%).

 The Kappa coefficient was estimated separately for dogs and cats (0.12 and 0.14 for dogs and cats, resp.). There was a little agreement between parasite detection by the hemoculture and PCR assay.

## 4. Discussion

According to the Secretariat of Health Surveillance of the Ministry of Health from Brazil, the area originally of risk of vector transmission of Chagas disease in country, currently covers 18 states, among which is São Paulo state [13].

In São Paulo state, Brazil, it is considered that Chagas disease transmission by *T. infestans* has been interrupted since the early 1970s. In the microregions of Avaré and Itapetininga cities, transmission was considered to be interrupted in 1974 because, since then, few triatomids, among those captured, were infected [14]. If a “residual transmission” occurred after these years, it must have taken place in a restricted area in the region of Sorocaba and in cities belonging to the microregions of Avaré and Itapetininga cities [15].

Godoy and Meira [15] studied evidence of vector transmission of *Trypanosoma cruzi* after 1983 in the houses and peridomiciliar areas of chagasic individuals residing in the region of Botucatu, SP, Brazil, and found that schoolchildren born from 1973 to 1983 did not show seropositivity after 1982, thus indicating the efficiency of the campaign and control against Chagas disease in this region.

However, although there is evidence that Chagas disease is controlled in the region of Botucatu, SP, Brazil, University Hospital of the Botucatu College of Medicine still provides care to many *T. cruzi *infected individuals diagnosed for blood donation as well as by the various specialities clinical and surgical from the hospital [5]. Furthermore, the high frequency of trypanosomatids infection in dogs belonging to individuals with chronic Chagas disease verified in the study of Lucheis et al. [5] could be pointing to a transmission cycle, whereby triatomines may be involved, although they were not been detected in the domiciles visited. Dogs are one of the main sources of household food for triatomids, such as *Triatoma infestans, Panstrongylus megistus,* and *Rhodnius neglectu *as well as humans and birds [16].

Even in face of the reduced occurrence of the disease in São Paulo state, Brazil, and the region of Botucatu, SP, Brazil, the 20% of positivity for *T. cruzi* shown by PCR in dogs from this municipality in this study confirm the findings by Lucheis et al. [5]. They studied a total number of 50 dogs belonging to 30 chronic chagasic individuals in the western region of São Paulo state, Brazil, and found that 34 (68%) dogs were positive to xenodiagnosis, 30 (60%) positive to hemoculture and 25 (50%) positive to PCR. The occurrence of 20% of animals infected with *T. cruzi* among the cats from Botucatu, SP, Brazil, evaluated in this study corroborates the results of another study conducted by Cardinal et al. [17], which showed positivity of 4.58% to PCR in 109 cats and concluded that they are also natural reservoirs for this protozoan. Hence, it can be speculated that the 20% of positivity to PCR for dogs and cats found in this study show that such species are exercising an important role as *T. cruzi* reservoirs in the region of Botucatu, SP, Brazil.

The chronic form of Chagas disease is characterized by a low level of parasitemia. Laboratory diagnosis in this phase can be performed by means of various techniques; however, all of them have limitations. Indirect parasitological tests, such as hemoculture, is a high-specificity test and presents limited sensitivity in the chronic form of the disease, due to a transitory and low parasitemia [18]. Their sensitivity increases when the volume and number of samples are larger, when the time between collection and processing is shorter, when the LIT medium is used for culture [11], and when blood samples are few manipulated [19].

In the hemoculture technique, several fields of blades are necessary for a definitive interpretation of a negative result. Observers must also recognize the rapid movements of trypanosomatids so that they are not confused with other protozoans, bacteria and fungi possibly present in the hemoculture [20]. Maybe this limitation can explain a negative sample for hemoculture and positive for PCR.

Furthermore, the accurate identification of *T. cruzi* is rather limited by the similarity to other trypanosomatids and other protozoa such as leishmanias and requires identification by means of a specific molecular test [21].

Luz et al. [22] reported 94% sensitivity using 30 mL of blood in LIT medium and the maximum time of 30 minutes for the test. The blood volume recommended for blood culture is 30 mL for humans [22]; however, in the case of the animals in this study, the collection of 5 mL–8 mL per animal was standardized in order to adapt to the dogs' and cats' weight and age variability. Perhaps a larger volume of inoculated blood could increase the sensitivity of hemoculture tests.

Minter-Goedbloed et al. [23] demonstrated that the number of positive results for hemoculture increased when the cultures were examined longer than six months of incubation. On the other hand, in the present study, the results showed that, of the seven positive samples among the animals from Botucatu, SP, Brazil, only one was diagnosed after 40 days of inoculation. This fact suggests that survival of *T. cruzi *in the LIT medium may be reduced over time instead of its multiplication.

Application of hemoculture can be restricted due to its low sensitivity and due to the long time period until the obtention of the final result [24], in addition to the fact that parasite's survival in the LIT medium may decrease over time.

Statistical analysis of the present study confirms the low sensitivity and low relative sensitivity of hemoculture and the high specificity and sensitivity of PCR in the detection of *T. cruzi*, thus corroborating the studies by Araújo et al. [10], who found 100% positivity for PCR and only 22% for blood culture from experimentally infected dogs.

As concerns the primers used in the present study, the detection limit of primers TCZ1/TCZ2 is at least 0.002 parasite/assay, which is higher than other primers. Also, their sensitivity is tenfold than other primers, such as P35/P36 [12].

Furthermore, the use of primers TCZ1/TCZ2 is able to detect a single parasite in a volume of blood of 0.1 mL. Such sensitivity is sufficient to detect infection both in the acute and chronic phase of Chagas disease [12].

In the present study, it is important to consider that PCR was performed from the blood culture and not directly from the blood. Due to the enrichment of LIT medium, it is possible to increase the number of parasites in the culture, compared with parasites found in blood; so, it is expected that sensitivity of PCR tends to be higher. Because of this, in this study the PCR technique was performed as a gold standard when evaluating the detection of *T.cruzi* by blood culture from dogs and cats.

## 5. Conclusions

Results showed that hemoculture technique, as determined by the Polymerase Chain Reaction (PCR) using primers TCZ1/TCZ2, presented low relative sensitivity. According to the results, we conclude that in this research dogs and cats in Botucatu, SP, Brazil, were exercising role of household reservoirs of *T. cruzi*, which reinforces the need for constant surveillance of this zoonosis in this region. Thus, it is necessary to evaluate how these animals became infected and if they can also act as sources of infection to humans.

## Figures and Tables

**Table 1:** Hemoculture and Polymerase Chain Reaction (PCR), using primers TCZ1/TCZ2, in dogs and cats from Botucatu-SP, Brazil.

PCR	Hemoculture				
	Positive *n* (%)		Negative *n* (%)		Total *n *(%)
	Male *n* (%)	Female *n* (%)	Male *n* (%)	Female *n* (%)	
Positive	1 (1)	0 (0)	8 (8)	11 (11)	20 (20)
Negative	3 (3)	3 (3)	29 (29)	45 (45)	80 (80)
	4 (4)	3 (3)	37 (37)	56 (56)	
Total *n* (%)	7 (7)		93 (93)		100 (100)
